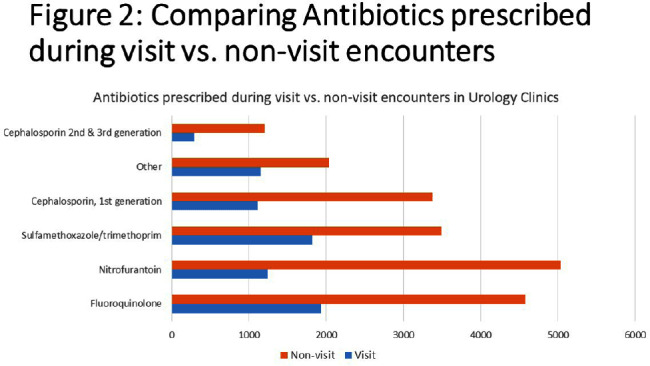# The Uncharted Patterns of Antibiotic Prescribing in Urology Ambulatory Practices: A Four-Year Analysis

**DOI:** 10.1017/ash.2024.103

**Published:** 2024-09-16

**Authors:** Sonal Munsiff, Kathleen Holt, Sucharu Ghosh, Ghinwa Dumyati

**Affiliations:** University of Rochester; Staff; University of the Pacific; University of Rochester Medical Center

## Abstract

**Background:** Urinary tract infections (UTIs) represent a prevalent indication for outpatient antibiotic usage, yet limited data exist regarding antibiotic prescriptions within urology specialties. This study aimed to assess antibiotic prescribing patterns in urology offices over a four-year period, providing insights for potential stewardship interventions. **Methods:** The analysis focused on antibiotic prescribing trends in adults between 2018 and 2021 during both visit and non-visit (e.g. telephone and chart messages) encounters across 15 ambulatory Urology clinics in an academic medical center in Western New York. Exclusions were made for antivirals, antiparasitics, antifungals, oral suspensions, selected non-UTI antibiotics, duplicate orders on the same day or week, and prescriptions exceeding 28 days. Prescriptions were categorized into single doses administered in the clinic and those prescribed for 2-28 days, with descriptive statistics and trend analyses conducted using SAS v9.14. **Results:** Over the four-year period, 54,282 prescriptions were analyzed. Of these, 26,944 (49.7%) were single doses administered in the clinic, predominantly for pre-procedure prophylaxis. The most commonly prescribed antibiotics for prophylaxis were fluoroquinolones (FQ) (47.5%), followed by ceftriaxone (19.2%), nitrofurantoin (13.2%), trimethoprim/sulfamethoxazole (8.6%), and gentamicin (4.2%). Among the 27,288 prescriptions for 2-28 days, 72.3% were from non-visit encounters, with 61.6% prescribed by advanced practice providers (APPs) (Figure 1). The mean number of prescriptions per patient was 2.07, with women receiving more prescriptions than men (2.39 vs. 1.88, P < 0.001). FQ remained the most commonly prescribed antibiotics during all encounters (23.7%), followed by nitrofurantoin (23.0%) (Figure 2). The antibiotic duration was longer for visit-based compared to non-visit-based prescriptions (mean 10 vs. 7 days, P < 0.001). Notably, there was a significant decrease in fluoroquinolone use between Q1 2018 and Q4 2019 for both male and female patients, followed by insignificant changes thereafter. **Conclusions:** Antibiotic use in urology outpatient settings is substantially underestimated if only prescriptions made during visit encounters are considered. More than two-thirds of prescriptions for 2-28 days were from non-visit encounters, with the majority originating from APPs. The average therapy duration exceeded guideline recommendations. Moreover, approximately half of the antibiotics were administered in the office for pre-procedure prophylaxis. To enhance antibiotic prescribing in these specialized clinics, interventions should focus on non-visit prescriptions and provide education for APPs, alongside adjustments to default durations in electronic orders. Further evaluation is essential to assess the appropriateness of single doses for pre-procedure prophylaxis.

**Figure:**
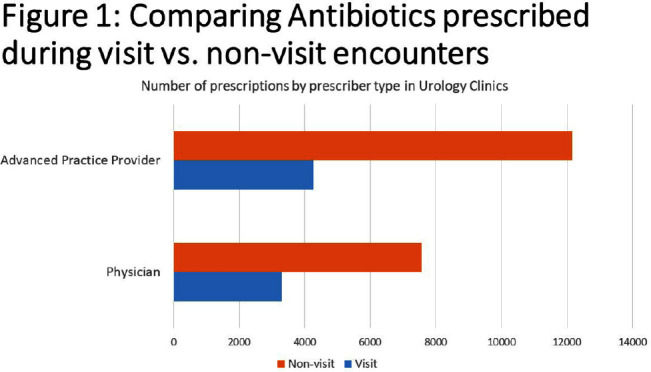

**Figure:**